# Cell and molecular biology: a new section joins the fight against cancer

**DOI:** 10.1038/bjc.2017.443

**Published:** 2017-12-12

**Authors:** Colin R Goding

**Affiliations:** 1 Ludwig Institute for Cancer Research, University of Oxford, Nuffield Department of Clinical Medicine, Old Road Campus Research Building, Headington, Oxford OX3 7DQ, UK

In 1971, the Nixon administration passed the US National Cancer Act and declared the ‘war on cancer’, launching an intensive campaign to find a cure. Forty-five years later, the US Congress passed the 2016 21st Century Cures Act with a similar aim. Between these two landmark funding initiatives, we have seen major changes in our understanding of cancer and the therapeutic approaches available.

These changes include the identification and cloning of oncogenes and tumour suppressors, the development of anticancer vaccines targeting hepatitis virus and more recently human papilloma virus, and the introduction of therapies targeted at specific signalling pathways deregulated in cancer. Whereas studies once might have focussed on examining the role and impact on disease progression of a single gene, the advent of high-throughput sequencing technologies now enables genome-wide analysis of genetic lesions. This in turn allows the evolution of the cancer genome during disease progression to be determined. Advances in understanding the complexities of the immune system have led to the development and application of immunotherapies designed to harness the power of the innate and adaptive immune system. Together with enhanced screening, imaging and more precise targeting of therapies, there is no doubt that substantial progress has been made in the war on cancer.

Yet, in many ways progress is less than we might have imagined. Resistance to targeted mono-therapies seems almost inevitable. For example, the early and dramatic success of inhibitors targeting BRAF in melanoma was met with great excitement. Yet, it quickly emerged that resistance usually occurs within a few months. More daunting was the realisation that different metastases within the same patient might contain cells with different mechanisms of resistance. The success and ultimate failure of BRAF-targeted therapy for melanoma provides a lesson relevant for many anticancer strategies. Even the current success of immunotherapy is tempered with the realisation that not all patients or tumours respond and resistance can emerge.

Despite these issues, the future holds substantial promise if only because we are now more aware of the extent of the challenges we face in the development of truly curative therapies. Whereas once tumours were considered to be largely a homogenous mass of cells, the extent of genetic and phenotypic heterogeneity is now widely appreciated, and we can begin to identify strategies to overcome the barriers heterogeneity poses to targeted and immunotherapies. Moreover, the advent of technologies, including intravital imaging (that enables the behaviour of individual cells within tumours to be visualised) and single-cell gene expression profiling, is revealing more detail about how the tumour microenvironment can shape cancer progression. Future success in the fight against cancer will be underpinned in part by our ability to decipher the impact of the complex and reciprocal interplay between genetics, epigenetics and the intratumour microenvironment that dictates treatment outcome.

One of the common threads linking past and future progress in the fight against cancer is the powerful combination of cell and molecular biology. Deciphering how gene function affects cell behaviour is crucial to our understanding of the molecular basis of cancer progression and therapy resistance. Genome-editing tools, the development of three-dimensional culture systems that more accurately reflect cell growth within tumours than standard two-dimensional culture, and the ability to use high-throughput genetic and drug-screening tools are likely to make a major impact on both our understanding of the disease and the identification of novel therapeutic vulnerabilities. Such tools will reveal how the complex intratumour microenvironment, which includes cancer cells, infiltrating immune cells and cancer-associated fibroblasts, effects the phenotypic transitions that represent key challenges to effective therapy.

Given the fundamental importance of this field to current and future cancer research and to the development of new and successful anticancer therapies, it is with great enthusiasm that we are launching a new Cell and Molecular Biology section in the *British Journal of Cancer*. We welcome increased submissions in this area including those that provide key insights into:

— The molecular mechanisms underpinning cancer progression;
— Mechanisms of drug and immunotherapy resistance;
— The tumour microenvironment;
— Cancer metabolism;
— Phenotypic heterogeneity;
— Dormancy;
— DNA damage repair pathways;
— Antitumour immunology;
— Preclinical models for cancer; and
— New methodologies.

By including a section dedicated to cell and molecular biology, we recognise the importance of this field to the future of cancer research. We also anticipate that articles covering the topics highlighted above will complement the existing, more clinically oriented, subject areas that have led to the *British Journal of Cancer* being recognised as one of the most-cited cancer journals.

